# In House Validated UHPLC Protocol for the Determination of the Total Hydroxytyrosol and Tyrosol Content in Virgin Olive Oil Fit for the Purpose of the Health Claim Introduced by the EC Regulation 432/2012 for “Olive Oil Polyphenols”

**DOI:** 10.3390/molecules24061044

**Published:** 2019-03-16

**Authors:** Maria Z. Tsimidou, Michaela Sotiroglou, Aspasia Mastralexi, Nikolaos Nenadis, Diego L. García-González, Tullia Gallina Toschi

**Affiliations:** 1 Laboratory of Food Chemistry and Technology, School of Chemistry, Aristotle University of Thessaloniki (AUTH), 541 24 Thessaloniki, Greece; michaelasotiroglou@outlook.com (M.S.); aspamastralexi@yahoo.gr (A.M.); niknen@chem.auth.gr (N.N.); 2 Instituto de la Grasa (CSIC), Ctra. de Utrera, km. 1, Campus Universitario Pablo de Olavide-Building 46, 41013 Seville, Spain; dlgarcia@ig.csic.es; 3 Department of Agricultural and Food Sciences, Alma Mater Studiorum-University of Bologna (UNIBO), Piazza Goidanich, 60, I-47521 Cesena (FC), Italy; tullia.gallinatoschi@unibo.it

**Keywords:** UHPLC, diode array detection, hydroxytyrosol, tyrosol, health claim, virgin olive oil, validation, European Commission Regulation 432/2012, olive oil phenols

## Abstract

An ongoing challenge in olive oil analytics is the development of a reliable procedure that can draw the consensus of all interested parties regarding the quantification of concentrations above the required minimum value of 5 mg of bioactive “olive oil polyphenols” per 20 g of the oil, to fulfill the health claim introduced by the European Commission (EC) Regulation 432/2012. An in-house validated ultra-high performance liquid chromatography (UHPLC) protocol fit for this purpose is proposed. It relies on quantification of the total hydroxytyrsol (Htyr) and tyrosol (Tyr) content in the virgin olive oil (VOO) polar fraction (PF) before and after acidic hydrolysis of their bound forms. PF extraction and hydrolysis conditions were as previously reported. The chromatographic run lasts ~1/3 of the time needed under high performance liquid chromatography (HPLC) conditions, this was also examined. Eluent consumption for the same piece of information was 6-fold less. Apart from being cost effective, a larger number of samples can be analyzed daily with less environmental impact. Two external curves, detection at 280 nm and correction factors for molecular weight difference are proposed. The method, which is fit for purpose, is selective, robust with satisfactory precision (percentage relative standard deviation (%RSD) values < 11%) and recoveries higher than 87.6% for the target analytes (Htyr, Tyr). Standard operational procedures are easy to apply in the olive oil sector.

## 1. Introduction

The European Union (EU) regulatory methods developed for olive oil characteristics are focused on authenticity and quality issues, including sensory attributes [1]. The same applies for the methods recommended by the International Olive Council (IOC) [2], Codex Alimentarius Committee [3], the United States Department of Agriculture (USDA) [4] and other relevant scientific bodies and food authorities. It is generally acknowledged that the regulatory requirements for this edible oil that is traded in different commercial categories (e.g., extra virgin, virgin, olive oil composed of refined olive oils and virgin olive oils) [5] are rather detailed and recently more criteria have been added to further ensure its quality, authenticity and consumer safety [6]. Most of the methods adopted by the European legislation have emerged from those recommended by the IOC activities [2]. This fact is justified as Spain, Greece, Italy, Portugal, France, Croatia and Slovenia, which are the major olive oil producing and consuming EU member states, pay a lot of attention to the protection of this important agricultural product for their national economies and collaborate in working groups under the auspices of the IOC since its establishment. The analytical requirements are currently expanded beyond the above-mentioned ones to address issues raised by non-compulsory labeling, such as the geographical indications, monovarietal origin, organic label, nutrition and health claims. The latest analytical challenge concerning olive oil is the one raised after the issuing of EC Reg. 432/2012 [7], which authorizes a health claim for those olive oils that contain at least a minimum concentration of endogenous “polyphenols” active for “the protection of blood lipids from oxidative stress”. No method was specifically suggested or adopted in the regulation for the measurement of the 5 mg of hydroxytyrosol and derivatives (e.g., oleuropein complex and tyrosol) per 20 g oil. This gap, together with some disagreements among scientists and authorities on which individual compounds among the numerous (more than 30) known olive oil phenolics [8] should be summed up, led the olive oil sector to limit the implementation of this claim, to avoid legal implications [9,10].

Technically speaking, there are hundreds of publications on the analysis of olive oil phenolic compounds, the most important of which are oleuropein and ligstroside derivatives, but no fully validated procedures are available so far, to our knowledge. The findings of these papers are frequently reviewed and critically discussed [11,12,13,14,15]. These compounds are mainly determined in the PF of the oil by liquid chromatography, capillary electrophoresis and gas chromatography, though they can be also determined in the intact oil. Among them, the IOC had adopted in 2009 [16] a procedure that is articulated by a PF extraction step, a separation protocol, ultraviolet (UV) detection at 280 nm and a rather ambiguous quantification approach. Soon after the health claim was issued, the IOC recognized the need for the development of a “fit for purpose” method suitable for addressing the analytical requirements in an open call [17]. Toward this direction some research groups published papers in peer review journals presenting more or less sophisticated approaches [18,19,20,21,22,23,24]. Most of them involve hydrolysis of the bound forms of Htyr and Tyr, and calculation of their total content in the free form after separation on a liquid or gas chromatographic column using diode array or flame ionization detection systems, respectively, because these are widely available in official and quality control laboratories [18,19,20,23,24]. The two most promising hydrolysis protocols used in these publications have been developed to address other issues long before the launching of the regulation. The one published by Mulinacci et al. [25] deals with hydrolysis of the bound forms in the polar extract of the oil, whereas the one by Romero and Brenes [26] deals with their hydrolysis directly in the oil. Some previous discussions pointed out the advantages of the first protocol [19]. In brief, analysis of phenolic compounds before and after hydrolysis in the PF provides multiple types of information that are otherwise lost. Moreover, hydrolysis is completed faster in the PF than in the intact oil by a factor of 3 times and generates less waste. In the present work, ultra-high performance liquid chromatography (UHPLC) is selected as a green and faster means for phenolic compounds separation, to speed up overall analysis time. This choice is in line with current trends in liquid chromatographic applications in the food sector [27]. Detection at 280 nm is adequate because only Htyr and Tyr are determined. Special attention is paid to factors affecting accuracy. The procedure was in house validated [28] and is proposed to the olive oil sector as a reliable fit for purpose method in line with guidelines recommended by Eurachem [29] and other relevant method standardization bodies.

## 2. Results and Discussion

### 2.1. Analytical Protocol

The overall analytical procedure involves: (i) PF extraction in line with the IOC [2]; (ii) hydrolysis of an aliquot of PF in accordance with Mulinacci et al. [25] as adopted by Mastralexi et al. [19]; and (iii) determination of Htyr and Tyr in the PF prior and after hydrolysis as described by Mastralexi et al. [19]. Extraction solvent, methanol:water, 80:20, *v*/*v*, was standardized in our previous work [30]. In this work parameters like dilution of oil in hexane or not and the effect of ultrasound bath technical characteristics are clarified. As shown in Table 1, oil dilution did not significantly influence the total phenol amount determined by the Folin-Ciocalteu (F-C) assay as equivalents of caffeic acid (CA), or the individual total Htyr and Tyr content determined by high performance liquid chromatography coupled to diode array detector (HPLC-DAD). The same was shown for the ultrasound technical characteristics (data not shown), which is recommended to be referred in the documents upon application.

Paiva-Martins and Gordon [31] examined the stability of certain olive and olive oil phenolic compounds (0.6 mM each component) in a ethanol:water 65:35, *v*/*v* solution at pH 3.5, 5.5 and 7.4 and found no loss for Htyr after storage for 48 h (37 °C, in the dark). Zafra et al. [32] examined the effect of freezing, refrigerating or room temperature to the stability of Htyr aqueous solutions (2.5, 25, 250 mg/L) prepared in Milli-Q water for a week and suggested storage under freezing conditions to avoid any degradation. Under the conditions (acidic pH, 80 °C, 2 h) applied by Mulinacci et al. [25], in the absence of the lipid matrix, Htyr was reported as stable, a finding also verified by Mastralexi et al. [19] at a similar concentration level. In the present study, the stability of Htyr in the presence of the lipid matrix under the above conditions was examined and discussed in § 2.2.2.

The UHPLC protocol validated herein had as its starting point the high performance liquid chromatography (HPLC) protocol proposed by the IOC as it was scaled down by Schneider [33]. Adjustments/modifications were then carried out for its adaptation to laboratory facilities. The column selected was recommended by the chromatographic system manufacturers as the type with the highest-pressure tolerance (100 MPa) among the array of available long (75 × 2.0 mm) columns commercialized by them and suitable for applications involving mobile phases with high water content [34]. The column is end-capped, with a high surface area of 500 m^2^/g, a carbon load of 22%; chemically stable at pH 2–7.5. The particle size was 1.6 μm, which is below the maximum limit (2 μm) proposed for UHPLC applications [35]. By trial, the flow rate was set at 0.45 mL/min and the column temperature at 35 °C in order to maintain back pressure values within the acceptable working range of the instrument and safeguard column performance. Sample injection was set at 3 μL (2.5-fold higher than that calculated using the ThermoFisher HPLC method transfer calculator [36]) to increase the sensitivity, without observing peak splitting. Autosampler temperature was maintained at 6 °C to assure stability of the analytes in the extracts/solutions analyzed in replicate.

**Figure 1:**
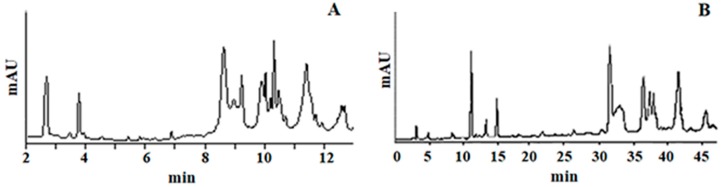
Profile of virgin olive oil (VOO) PF using (**A**) ultra-high performance liquid chromatography coupled to diode array detector (UHPLC-DAD) and (**B**) high performance liquid chromatography coupled to diode array detector (HPLC-DAD).

To examine the separation efficiency of UHPLC, the same virgin olive oil (VOO) PF fraction was also analyzed using the aforementioned IOC HPLC protocol [16]. The recorded profiles were compared and it was shown that UHPLC provided a similar type of separation (Figure 1) within 1/3 of the time needed to use HPLC.

Elution of Htyr and Tyr was achieved in less than 5 min. Taking into account environmental concerns, the mobile phase consumption was reduced almost 6-fold for the same piece of information. As a result, 48 complete runs can be achieved within 24 h using this UHPLC protocol, whereas using HPLC the number is only 17.

Because only the two analytes are quantified after hydrolysis, the quantification approach proposed by Mastralexi et al. [19] was adopted for the determination of total Htyr and Tyr content. As a consequence, the Htyr and Tyr amount corresponding to their bound forms is first calculated and then a correction factor (Htyr, 2.2; Tyr, 2.5) is used to account for the mass difference.

### 2.2. Method Validation

#### 2.2.1. Specificity

Resolution between the two peaks corresponding to the target analytes, Htyr and Tyr, was >>1 (~3.63). Peak purity determination using the diode array detector (DAD) showed that a single analyte could be assigned to each peak in the VOO PF before and after hydrolysis. This was further verified by matching the corresponding spectra to those of pure standards analyzed under the same conditions. Coupling to a fluorescence detector (FLD) under appropriate conditions of excitation showed two single fluorescent peaks assigned to the target analytes.

#### 2.2.2. Stability of the Analytes

##### Stability of Standard Solutions

Stock standard solutions of 1000 mg/L of Htyr and Tyr, respectively, were prepared in methanol:water, 50:50, *v*/*v* and stored at −18 °C for a period of one year. On four separate occasions, the stock solutions were warmed up to room temperature and working solutions of 20 and 80 mg/L (Htyr), 60 and 80 mg/L (Tyr) were prepared and measured. The percentage relative standard deviation (%RSD) values for Htyr analyzed at these two concentrations were 10.1 and 3.8%, whereas for Tyr were 6.5 and 2.8%, respectively.

Stability of Htyr and Tyr in working solutions of 10, 20 and 40 mg/L—prepared by dilution from the stock solution—was examined on four different days within a week. On the basis of the recorded areas, the %RSD values were 4, 1 and 1% (Htyr) and 4, 1 and 2% (Tyr), respectively, suggesting that the solutions were stable. Such an observation is in accordance with the short-term stability study of Zafra et al. [32] who, using UHPLC-MS, found out that storage at −20 °C for a week did not affect the signal of Htyr aqueous solutions prepared in the range 2.5–250 mg/L.

##### Stability of Spiked Samples

**Figure 2:**
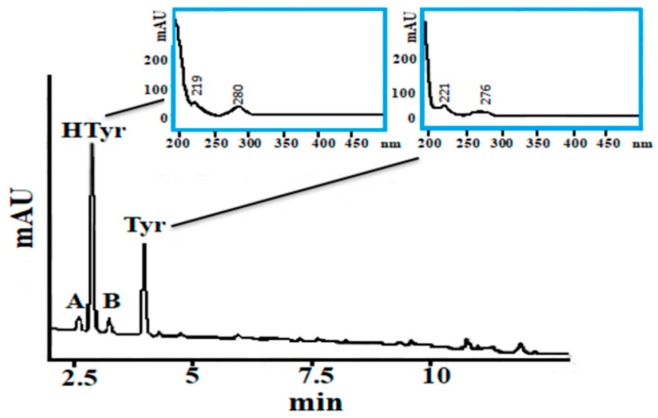
UHPLC-DAD recorded profile at 280 nm of the PF of a VOO sample after hydrolysis (the UV spectra corresponding to the peaks attributed to Htyr and Tyr, respectively, are shown in the inserts).

When the acidic hydrolysis conditions were applied to the PF of a VOO sample, the formation of two new peaks (A and B) were observed in the chromatogram at 280 nm, one eluting before and one after Htyr peak (Figure 2).

The relative retention times of these two new peaks A and B were 0.93 and 1.14 toward Htyr, respectively. These two peaks absorbed at 280 nm but did not fluoresce under the selected conditions applied for the two analytes. Peak A and B formation from Htyr was verified when refined olive oil (ROO), free of Htyr or Tyr, was spiked with 2.5 mg/20 g oil of Htyr or Tyr in separate experiments. The UV spectrum of peak A had an absorption maximum at 363 nm, which might be attributed to a quinone derivative of Htyr [37]. The unknown compound under peak B had similar UV spectra to Htyr but with a UV absorbance maximum at 269 nm instead of 280 nm. Spiking of the hydrolysate with 3,4-dihydrophenylacetic acid (DHPA), a Htyr oxidation product that can be formed upon the presence of hydrogen peroxide and iron [37] and is expected to elute soon after Htyr, did not match with the peak B elution time. Under acidic conditions and heating, examination of the PFs obtained from spiked ROO aliquots (ROO-1 to ROO-3) with known amounts of Htyr and Tyr (see § 3.8) on *intra*- (*n* = 15) and *inter*- (*n* = 45) day basis, indicated a certain instability of Htyr. Application of the protocol to 30 VOO PFs revealed the frequent formation of peaks A and B. Frequency distribution of the calculated relative area (%) for peak A, Htyr or B in the tested PFs showed that only in 10% of the samples the corresponding value for peak A or B was ≤5%. Thus, the two extra peaks were taken into account in the calculation of [Htyr] after hydrolysis in the following analyses. Due to the fact that peaks A and B do not fluoresce, the fluorescence detector (FLD) is not recommended for the purpose of the method.

#### 2.2.3. Linearity

Linearity was examined by the external standard method for a wide range of concentrations of Htyr and Tyr standard solutions. A linear response up to 100 μg/mL was evidenced for Htyr and up to 120 μg/mL for Tyr, (that is up to 500 and 600 ng injected, respectively). The coefficient of determination (*r*^2^) in both cases was ≥0.996. Moreover, the calculated sum of squares (SS) and mean square (MS) values for the two regression lines were higher than those of the residuals. Such an observation, combined with the high *F* values and low *p* values, supported the linear relationship within the tested range [38].

The upper limit for Htyr was comparable to that reported in the literature by Godoy-Caballero et al. [39], who used a rapid resolution LC system coupled to DAD for the determination of phenolic compounds in VOO and a rapid resolution Zorbax Eclipse XDB-C18 column (50 × 4.6 mm, 1.8 μm, Agilent Technologies, Palo Alto, CA, USA). In the case of Tyr, the respective limit was higher. More specifically, these authors provided as upper concentration values 51.5 and 53.0 µg/mL for Htyr and Tyr respectively, but with an injection volume of 10 µL (that is up to 515 and 530 ng injected, respectively). Celano et al. [40] in a recent publication aiming at getting further insight in the analysis of secoiridoids of VOO, examined linearity for Htyr and Tyr by UHPLC-UV in the range 5–200 µg/mL without providing the injection volume, which makes difficult to compare the actual analyte mass introduced onto the column used (100 × 2.1 mm, 2.6 µm column, Kinetex C18, Phenomenex, Bologna, Italy) with our data.

#### 2.2.4. Limit of Detection (LOD) and Limit of Quantification (LOQ)

LOD and LOQ values estimated for Htyr were 0.15 and 0.45 μg/mL (or 0.45 and 1.5 mg/kg oil) and for Tyr, 0.06 and 0.19 μg/mL (or 0.18 and 0.57 mg/kg oil). These findings support that the method is equally sensitive to other UHPLC ones for VOO phenol analyses using the same type of detection. Thus, Godoy-Caballero et al. [39] reported values for LOD/LOQ, of 0.4/1.0 μg/mL for Htyr, 0.08/0.3 μg/mL for Tyr and Celano et al. [40] 0.5/1.0 μg/mL and 1.4/2.5 μg/mL, respectively). Godoy-Caballero et al. [39] calculated LOD on the basis of the Long and Winefordner criterion [41] and then LOQ as 3.33 × LOD, whereas Celano et al. [40] calculated the corresponding values by extrapolation of the concentrations resulting in a signal to-noise ratio (S/N) of 3 and 10. The method is less sensitive to those coupled to MS detection, since the calculated LOD value, with minor exceptions, was ~10-fold higher [39,42,43,44]. The LOQ values for Htyr were ~3–9-fold lower with MS detection for Htyr, but this was not always the case for Tyr. Specifically, in some publications a 1.6 or 4.9-fold higher LOQ value has been found [39,42], whereas in others 2.9 and 6.3-fold lower values were found [43,44]. Discrepancies may be partially due to the different approaches followed for the calculation of these parameters. For this reason, no further comparisons were made with our values, which were calculated as recommended by the International Conference on Harmonization (ICH) [45].

#### 2.2.5. Precision

*Intra*- and *inter*- day measurement repeatability was examined for the two analytes. The *intra*- day %RSD values were in the range 0.1–3.7 for Hytr and 0.1–3.4 for Tyr. Those of *inter*-day were in the range 0.2–5.7 and 0.3–5.6, respectively. Higher values were obtained when solutions containing lower concentrations of analytes were measured. The repeatability of the measurement was excellent, as the values for the different levels of concentration were lower than those set by the Association of Official Agricultural Chemists, (AOAC) (%RSD limit: 15% for 0.1 mg/kg; 11% for 1 mg/kg; 7.3% for 10 mg/kg; 5.3% for 100 mg/kg) [46]. Godoy-Caballero et al. [39] reported %RSD *intra*-day values of 3.0 and 0.6 for Htyr and Tyr, respectively and 3.7 and 2.3 *inter*-day for a single solution containing 5 µg/mL (50 ng injected), which have been estimated in terms of peak areas instead of concentration.

Repeatability of the method was examined via: (i) analysis of the PFs before and after hydrolysis of three VOOs (VOO-4, -5 and -6) containing 296, 155 and 365 mg CA/kg, respectively; and (ii) analysis of the PFs before and after hydrolysis of three aliquots of ROO spiked with both analytes at three different levels of concentration (see § 3.8). Data are presented in Table 2.

The %RSD values for both Htyr and Tyr content were found to be <11% and, therefore, were considered acceptable [46]. The findings were comparable to those of Becerra-Herrera et al. [43], who using tandem mass spectrometry (MS/MS) provided %RSD values in the range 4–11.8% for a single concentration level (12 mg/kg) spiked in ROO. Their values were considerably lower than those of Alcarón-Flores [47], who also used MS/MS but spiked an olive oil sample with concentration levels of 0.5 and 1.0 mg/kg and reported values in the range ~9–26%. In the latter case, the low concentration levels used and the selection of a sample for spiking which already contained the analytes may possibly explain the high %RSD calculated values.

#### 2.2.6. Recovery Studies

The estimated *intra*- (*n* = 15) and *inter*- (*n* = 45) day recovery values examining the PFs from ROO-1, -2 and -3 samples (see § 3.8) after hydrolysis were 95–121% (mean = 107.8%) for Htyr and 90–115% (mean = 99%) for Tyr, respectively. A VOO containing mainly bound forms was spiked with 1.5 mg/20 g of Htyr and 3.5 mg/20 g of Tyr and recovery found was 117% for Htyr and 96.7% for Tyr, respectively. When aliquots of ROO (ROO-4) were spiked with a specific amount of PF of known concentration in total Htyr (1.5 mg/20 g oil) and Tyr (3.5 mg/20 g oil), the estimated recovery for the two analytes was 105% and 87.6%, respectively. In all cases, these values were satisfactory considering the range 80–110% proposed by AOAC [46]. Celano et al. [40] reported recoveries for both Htyr and Tyr between 98 and 106% without providing further details on the levels of concentrations used. In spiked ROO at 4 mg/kg, Becerra-Herrera et al. [43] obtained a recovery of 101.8 and 110.5 for Htyr and Tyr respectively, with MS detection. On the other hand, Alcarón-Flores, et al. [47] reported satisfactory values for Htyr (99 and 91% for 0.5 and 1.0 mg/kg respectively), whereas for Tyr were low (75 and 82% for 0.5 and 1.0 mg/kg respectively). As previously mentioned, though the authors applied MS detection, the levels of concentrations used were low and the substrate was an olive oil, which intrinsically contained the target analytes.

#### 2.2.7. Robustness

During routine analysis, the analyst has to change columns, preferably from the same batch of a particular packing material, provided by the same supplier. Thus, in the present study, calibration curves for the two analytes were constructed on 2 different column batches (batch A, one column: batch B, two columns) of the same packing material (Shim-pack XR-ODS III) purchased from the same supplier at different time points. The *r*^2^ values were >0.994 (Table 3). The slopes of the curves were first compared by applying a two tailed student’s t test [48]. Statistical differences were found in all cases. For the same batch, differences in absolute t values from the critical ones were ~1.5-fold higher for this statistic. Comparison of the calculated concentrations for standard solutions containing 10, 40 and 80 μg/mL of Htyr or Tyr showed that differences using the three curves were not more than 1.1-fold (calculations not shown).

The effect of the analyst on method performance was examined by two different trained analysts. Analysis of 5 VOOs showed some statistically significant differences (Table 4) but the magnitude of the measured values was comparable.

#### 2.2.8. Implementation of the In-House Validated UHPLC to VOOs

The in-house validated UHPLC protocol was implemented to a set of randomly selected VOOs. The same PFs obtained were analyzed in parallel by HPLC-DAD following the IOC [16] protocol of analysis using two different PF extraction procedures. The results are shown in Table 5 and Table 6.

Comparison of analytical results obtained with UHPLC-DAD and HPLC-DAD showed statistically significant differences (paired samples *t*-test) in almost all cases. UHPLC provided higher values for total Htyr and Tyr content for 6 out of the 8 samples, compared to those by HPLC. However, the magnitude of derived values was similar, as for 6 out of 8 samples the Htyr and Tyr contents were only 1.03 to 1.39-fold higher. For Htyr the difference was lower than 1 mg/20 g oil for all samples, whereas for Tyr the difference was lower than 1 mg/20 g oil for 6 out of 8 samples. The same samples were then analyzed with the same UHPLC and HPLC protocols after a different extraction procedure of PF [19] and hydrolyzed as before. Data shown in Table 6 suggested that there is no a typical trend for higher values using UHPLC. What can be said, though, is that the two chromatographic techniques provide rather commutable data. It can also be clearly observed that the F-C assay cannot support the health claim.

## 3. Materials and Methods

### 3.1. Chemicals

Htyr (≥98%) was purchased from Extrasynthèse (Genay, France) and Tyr (≥98%) from Alfa Aesar GmdH & Co KG, (Karlsruhe, Germany), CA (98%) and DHPA (98%) were purchased from Sigma-Aldrich Chemie GmbH (Steinheim, Germany). Analytical-grade methanol, HPLC grade methanol, acetonitrile and water were obtained from ChemLab (Zeldegem, Belgium). Phosphoric acid (≥85%) and sulphuric acid (95–98%) were supplied by Sigma-Aldrich (Steinheim, Germany). Sodium carbonate anhydrous and Folin-Ciocalteu (F-C) reagent were obtained from ChemLab (Zeldegem, Belgium). Polyvinylidene fluoride (PVDF) membrane filters (0.22 µm) were from Schleicher & Schuell, (Dassel, Germany).

### 3.2. Samples

Refined olive oil (ROO) was kindly provided by Elais Unilever S.A. (Piraeus, Greece). Virgin olive oil (VOO) samples were provided by local producers, who guaranteed their authenticity. All of the samples fulfilled the limits of the category extra virgin olive oil (EVOO) in terms of free acidity, peroxide value and K_232_ and K_270_ values [1].

### 3.3. Instrumentation

Chromatographic analyses were carried out on: (i) a Shimadzu Nexera X2 UHPLC System, (Shimadzu Corporation, Kyoto, Japan) equipped with a LC-30AD pump, SIL-30AC autosampler (50 μL loop), a CTO-20AC column oven, a UV-visible diode array SPD-M30A detector (temperature controlled semi-micro flow cell of 2.5 μL) coupled to a RF-20AXS fluorescence detector (temperature controlled semi-micro flow cell of 3 μL); and (ii) a HPLC system equipped with a P4000 pump (Thermo Separation Products, San Jose, CA, USA), a Midas autosampler (Spark, Emmen, The Netherlands), a UV 6000 LP diode array detector (Thermo Separation Products). Lab Solution ver. 5.86 software (Shimadzu Corporation, Kyoto, Japan) was used for UHPLC data acquisition and analysis, whereas Chrom Quest software (version 3.0, Thermo Separation Products) was used for HPLC data. Other equipment included a spectrophotometer UV-1601 (Shimadzu Co., Kyoto, Japan) with UVProbe 2.33 (Shimadzu Co., Kyoto, Japan) data handling software. For sample preparation an IKA MS3 digital vortex (IKA, Staufen im Breisgau, Germany), an ultrasound bath Elmasonic S 30 (H), (Elma Schmidbauer GmbH, Singen, Germany) [37 kHz; 80 W; Unit outer dimensions W/D/H, 137/100/241, mm/mm/mm, Capacity: 2.75 L] and a ultrasound bath ELMA transonic ts 420 [35 kHz; 60 W; Unit outer dimensions W/D/H, 137/100/151, mm/mm/mm, Capacity: 1.75L], a HBA 200 benchtop centrifuge (Hettich Instruments LP, Tuttlingen, Germany) and a WB 3015 water bath (Bioline Scientific, Athens, Greece).

### 3.4. Preparation of Standard Solutions

Standard stock solutions of Htyr and Tyr at a concentration of 500 mg/L (low concentration range) and 1000 mg/L (intermediate to high concentration range) were prepared in methanol:water 1:1, *v*/*v*. Aliquots were further diluted with the same solvent mixture to prepare a series of calibration working solutions for each analyte in the range 0.5–100 µg/mL. Stock solutions were kept in dark volumetric flasks at −18 °C and warmed up to room temperature before use. Working solutions were prepared daily.

### 3.5. Sample Preparation

#### 3.5.1. Extraction of the Polar Fraction (PF)

VOO PF was prepared as described in the IOC protocol [16] without the addition of internal standard. In brief, 2.0 g of VOO were accurately weighted in a 10 mL screw-cap tube (15 mL). Then, 1 mL of methanol:water, 80:20 *v*/*v* was transferred to the previously weighed sample. The tube was sealed and the sample was vortexed for exactly 30 s. Then, 5 mL of methanol:water, 80:20 *v*/*v* were added and the sample was further vortexed for exactly 1 min. Extraction was carried out in an ultrasonic bath for 15 min at room temperature. After centrifugation at 5000 rpm for 25 min the supernatant phase was collected. Extraction was carried out in triplicate (unless otherwise stated). The isolated PFs were combined into a representative one, which was filtered through 0.22 µm PVDF membrane before injected onto the chromatograph.

#### 3.5.2. Acidic Hydrolysis

The procedure of Mulinacci et al. [25] as adopted by Mastralexi et al. [19] was followed. In brief, an aliquot (200 μL) of PF was mixed with 200 μL of a 1 M H_2_SO_4_ solution. The mixture was maintained in a water bath at 80 °C for 2 h. The procedure was carried out in triplicate. Each hydrolysate was then diluted with 200 μL of the extracting solvent (methanol:water, 80:20 *v*/*v*). The three replicates were combined to obtain a representative hydrolysate which was filtered through 0.22 µm PVDF membrane before injected onto the chromatograph.

### 3.6. Folin Ciocalteu Assay

F-C assay was applied according to Nenadis et al. [30]. Measurements were carried out at 725 nm after 1 h reaction period. CA was used as an external standard and results were expressed as mg CA/20 g of oil. The determination for each extract was performed in triplicate.

### 3.7. Chromatographic Conditions

UHPLC: The chromatographic analyses were carried out on a 75 × 2.0 mm, 1.6 μm Shim-pack XR-ODS III Shimadzu (Shimadzu, Kyoto, Japan). Mobile phase consisted of water (0.2% phosphoric acid) (A), methanol (B) and acetonitrile (C) at a flow rate 0.45 mL/min. The gradient elution program was as follows: 0–11 min, 2–25% B and 2–25% C; 11−13 min, 25–30% B and 25–30% C; 13−17 min, 30–50% B and 30–50% C; 17–20 min, 50% B and 50% C; 20–20.5 min, 50–2% B and 50–2% C; 20.5–28 min 2% B and 2% C. Equilibration was from 28 to 30 min. Column temperature was maintained at 35 °C and that of the sample at 6 °C. Injection volume for PF or its hydrolysate was 3 µL and for standard solutions 5 µL. Chromatograms were recorded at 280 nm (UV) and λ_exc._ 280 nm, λ_em._ 320 nm (FL).

HPLC: The chromatographic analyses were carried out on a 250 × 4.6 mm i.d., 5 μm Nucleosil 100, C18 column (MZ-Analysentechnik GmbH, Mainz, Germany). The mobile phase consisted of water (0.2% phosphoric acid) (A), methanol (B) and acetonitrile (C) at a flow rate 1.0 mL/ min. The gradient elution program was as described by IOC [16]. The injection volume for PF or its hydrolysate was 20 µL and for standard solutions, 10 µL. Chromatograms were recorded at 280 nm (UV).

Htyr and Tyr prior to and after hydrolysis were quantified at 280 nm using the respective external calibration curves (see § 3.4). The total amount of Htyr and Tyr is calculated as the sum of their free and bound forms after correction for molecular mass differences between free and bound forms. Correction factors are introduced in the quantification of individual total Htyr (2.2) and Tyr (2.5), which are obtained by dividing the mean molecular mass of the most known bound forms of Htyr and Tyr (343 amu) by the molecular mass of Htyr (154 amu) and Τyr (138 amu), respectively. The bound forms considered were: tyrosol glucoside (300.30 amu), aldehydic form of ligstroside aglycone (362.12 amu), dialdehyde form of ligstroside aglycone (304.34 amu), aldehyde form of oleuropein aglycone (378.37 amu), oleuropein aglycone (378.37 amu), (carboxymethylated) dialdehyde form of oleuropein aglycone (378.37 amu), (decarboxymethylated) dialdehyde form of oleuropein aglycone (320.34 amu), (decarboxymethylated) aldehyde form of oleuropein aglycone (320.34 amu).

### 3.8. Method Validation

Method validation was carried out according to the Eurachem Guide [29]. The method was tested for specificity, linearity, detection limit and limit of quantification, accuracy, precision, robustness and applicability.

Specificity of the method for the fluorescent target analytes (Htyr, Tyr) was tested using the peak purity function of the diode array detector after analysis of VOO PFs prior to and after acidic hydrolysis.

Linearity of the calibration curves was examined in the concentration range of 0.05–100 for Htyr and 0.05–140 μg/mL for Tyr. Each of the eight concentrations used was analyzed in quintuplicate [29]. Calibration curves were constructed on three consecutive days using the least squares method [49]. After fitting the data, the residuals plots were examined for possible patterns. Analysis of Variance (ANOVA) was also carried out and the values of the SS and MS for both the regression and the residuals were examined, along with the *F* and *F* significance values (better known as *p* value) to establish a strong linear relationship [38].

For the standard compounds, the limits of detection (LOD) and quantification (LOQ) were calculated as 3.3 × s_B_/b and 10 × s_B_/b, respectively [45], where b is the slope of the curve and s_B_ corresponds to s_y/x_ = SQRT[Σi(yi − ŷi)^2^/(*n*−2) − 1], where yi is the experimental value and ŷi the predicted one from the calibration curve [50]. To determine the LOD and LOQ values, and in order to avoid the influence of the high concentrations, linear curves based on low levels of both analytes (0.05, 0.1, 0.2, 0.4 and 0.8 μg/mL) were constructed [51]. Each standard solution was injected 5 times.

The precision of the method was tested as follows. Standard solutions of both Htyr and Tyr in the concentration range 0.5–100 μg/mL were measured 5 times *intra*-day and analyzed on three consecutive days aiming to evaluate the repeatability of the measurement (% relative standard deviation, %RSD). The repeatability of the method was then examined via analysis of the PFs of three VOOs using the F-C assay [19]. The samples were measured 5 times *intra*-day and also analyzed on three consecutive days. In addition, aliquots of ROO spiked with varying concentrations of both analytes (5 replicates) were analyzed in triplicate *intra*-day and on three consecutive days. The spiked levels used were 1.0, 1.5 and 2.5 mg/20 g oil for Htyr and 1.5, 3.5 and 5 mg/20 g oil so that the samples analyzed to contain a total amount of 2.5, 5.0 and 7.5 mg/20 g oil. The relative ratio of the two analytes was selected according to information provided by Tsimidou et al. [10].

Accuracy of the method was examined by recovery experiments. More specifically, a set of PFs obtained from ROO aliquots spiked with the target analytes (5 replicates, 3 concentration levels, see previous paragraph) were chromatographed prior and after acidic hydrolysis in triplicate. The examination was made *intra*-day and on three consecutive days. In addition, a VOO containing mainly bound forms of the analytes (total Htyr, 0.48 mg/20 g oil; total Tyr, 0.83 mg/20 g oil), was spiked with the target analytes at one concentration level (Htyr: 1.5 mg/20 g oil, Tyr: 3.5 mg/20 g oil). Aliquots were prepared in quintuplicate. Each of them was chromatographed in triplicate. For correction the same procedure for PF preparation was followed without spiking with the target analytes. Recovery of bound Htyr and Tyr forms was also examined via spiking aliquots of ROO (*n* = 5) with the PF of a VOO at one concentration level of total Htyr and Tyr (~5 mg/20 g oil). The spiked samples were analyzed in triplicate as well.

Robustness was examined by constructing calibration curves for the two analytes on two different batches of the same packing material (batch A, one column; batch B, two columns) purchased from the same supplier at different time points. Furthermore, the PFs of 5 VOOs were analyzed by two different trained analysts on the same equipment. Extraction and hydrolysis of the extract and UHPLC analyses were accomplished within 24 h by each analyst, using the same batches of reagents and solvents. For each sample, run in triplicate, Analyst 2 carried out chromatographic analysis after Analyst 1.

### 3.9. Statistical Analyses

Data were expressed as mean ± standard deviation or as % relative standard deviation (%RSD) values. The data were statistically analyzed using the SPSS 14.0 software (SPSS Inc., Chicago, IL, USA). All other mathematical data treatment was carried out with Microsoft Excel, 2016 and the analysis tool Pak (Microsoft Corp., Redmond, WA, USA).

## 4. Conclusions

A UHPLC-DAD method fit for the purpose of the health claim introduced by the EC Regulation 432/2012 for “olive oil polyphenols” has been developed and in-house validated for the first time. The chromatographic run lasts ~1/3 of the time needed under HPLC conditions and eluent consumption to obtain the same piece of information is 6-fold less. Except for being cost effective, a larger number of samples can be analyzed daily with less environmental impact. The use of UHPLC after fourteen years of commercialization has been expanded in all types of analytical laboratories, whereas instrumentation cost is no higher than that of conventional HPLC. Consequently, it is realistic that due to its simplicity and rapidity the proposed analytical protocol may be applied in VOO quality control.

## Figures and Tables

**Table 1:** Effect of dilution of oil in hexane (1:1, *v*/*v*) prior to polar fraction (PF) extraction on the total phenol content (mg CA/kg oil) and total hydroxytyrsol (Htyr) and tyrosol (Tyr) content (mg/20 g oil).

Samples	Total Phenol Content *	Total Htyr Content	Total Tyr Content
	mg/20 g Oil		
VOO-1_IOC_	4.74 ± 0.47 ^b^	3.79 ± 0.32 ^a^	2.39 ± 0.35 ^a^
VOO-1_IOCmodified_	5.37 ± 0.32 ^a^	3.94 ± 0.31 ^a^	2.25 ± 0.20 ^a^
VOO-2_IOC_	10.97 ± 0.42 ^b^	3.80 ± 0.10 ^a^	5.60 ± 0.18 ^a^
VOO-2_IOCmodified_	11.62 ± 0.32 ^a^	3.77 ± 0.03 ^a^	5.67 ± 0.17 ^a^
VOO-3_IOC_	5.96 ± 0.20 ^a^	2.45 ± 0.18 ^b^	2.99 ± 0.21 ^b^
VOO-3_IOCmodified_	5.71 ± 0.40 ^a^	2.81 ± 0.04 ^a^	3.50 ± 0.02 ^a^

* Determined by the Folin-Ciocalteu (F-C) assay; Values are means ± standard deviation; (*n* = 3); each pair of values per sample in the same column bearing different lowercase letters as superscripts are statistically different at *p* < 0.05 (*t*-test); International Olive Council (IOC) modified means dilution of the oil in hexane prior to PF extraction.

**Table 2:** *Inter*- and *intra*- day repeatability of the method examined via: (i) analysis of VOOs; and (ii) refined olive oil (ROO) aliquots spiked with three different concentrations of Htyr and Tyr.

Sample	*Intra*-Day				*Inter*-Day			
	Total Htyr Content (mg/20 g)	%RSD	Total Tyr Content (mg/20 g)	%RSD	Total Htyr Content (mg/20 g)	%RSD	Total Tyr Content (mg/20 g)	%RSD
VOO-4	3.11 ± 0.32	10.4	5.29 ± 0.31	5.9	3.03 ± 0.31	10.2	5.17 ± 0.14	2.7
VOO-5	1.76 ± 0.11	6.0 (*n* = 5)	3.37 ± 0.12	3.6 (*n* = 5)	1.75 ± 0.11	6.0 (*n* = 15)	3.22 ± 0.19	5.8 (*n* = 15)
VOO-6	5.55 ± 0.18	3.2	3.76 ± 0.05	1.2	5.66 ± 0.12	2.1	3.66 ± 0.13	3.6
ROO-1 *	0.95 ± 0.06	6.4	1.73 ± 0.04	2.5	0.97 ± 0.07	7.4	1.67 ± 0.12	6.9
ROO-2 *	1.74 ± 0.05	2.6 (*n* = 15)	3.22 ± 0.10	3.0 (*n* = 15)	1.79 ± 0.08	4.4 (*n* = 45)	3.28 ± 0.15	4.6 (*n* = 45)
ROO-3 *	2.67 ± 0.12	4.4	4.48 ± 0.15	3.4	2.71 ± 0.11	4.2	4.58 ± 0.23	5.1

Values are means ± standard deviation; * spiked levels 1.0, 1.5 and 2.5 mg/20 g oil for Htyr and 1.5, 3.5 and 5 mg/20 g oil for Tyr, so that samples to contain as sum of total Htyr and Tyr 2.5, 5 and 7.5 mg/20 g oil.

**Table 3:** Calibration curves for Htyr and Tyr (μg/mL) constructed using two batches of the same packing material (three columns in turn of use).

Analyte	Working Range		y = a + bx	b ± SD	a ± SD
	μg/mL	*r* ^2^			
280 nm					
1st column, batch A					
**Htyr**	0.5–100	0.996	y = 11,176.4x + 8415	11,176.4 ± 110.6	8415 ± 5323
**Tyr**	0.5–100	0.996	y = 6109.7x + 4331	6109.7 ± 81.2	4331 ± 4268
2nd column, batch B					
**Htyr**	0.5–100	0.997	y = 11,817.4x + 17,025	11,817.4 ± 135.5	17,025 ± 6520
**Tyr**	0.5–100	0.994	y = 7261.9x + 8640	7261.9 ± 119.6	8640 ± 6288
3rd column, batch A					
**Htyr**	0.5–100	0.999	y = 10,954.8x − 751	10,954.8 ± 62.7	751 ± 3019
**Tyr**	0.5–100	0.998	y = 6187.8x + 15,359	6187.8 ± 62.9	15,359 ± 3308

**Table 4:** Determination of Htyr and Tyr content of 5 VOOs samples by two trained analysts using the same equipment and analytical conditions.

Analyst	Sample	Total Htyr Content	Total Tyr Content
		(mg/20 g Oil)	
1	VOO-4	2.96 ± 0.33 ^a,^*	5.08 ± 0.26 ^a,^*
2		3.11 ± 0.32 ^a,^*	5.29 ± 0.31 ^a,^*
1	VOO-5	1.85 ± 0.02 ^a^	3.45 ± 0.01 ^a^
2		1.63 ± 0.03 ^b^	2.91 ± 0.02 ^b^
1	VOO-6	5.76 ± 0.01 ^a^	3.72 ± 0.01 ^a^
2		5.68 ± 0.03 ^a^	3.59 ± 0.03 ^b^
1	VOO-7	4.52 ± 0.46 ^a^	5.70 ± 0.02 ^a^
2		4.08 ± 0.04 ^a^	3.86 ± 0.02 ^b^
1	VOO-8	1.14 ± 0.01 ^a^	1.54 ± 0.01 ^a^
2		1.01 ± 0.02 ^b^	1.17 ± 0.02 ^b^

Values are means ± standard deviation; *n* = 3; (* *n* = 5); Values bearing different lowercase letters as superscripts for each analyte per sample determined by the two analysts are statistically different at *p* < 0.05 (paired *t*-test).

**Table 5:** Determination of total phenol, total Hytr, total Tyr content (mg/20 g oil) for VOO samples analyzed by UHPLC-DAD and HPLC-DAD.

Samples	F-C Assay	UHPLC-DAD		HPLC-DAD	
	Total Phenol Content	Total Htyr Content	Total Tyr Content	Total Htyr Content	Total Tyr Content
	(mg CA/20 g Oil)	(mg/20 g Oil)			
VOO-1	4.74 ± 0.47	4.33 ± 0.03 ^a^	2.64 ± 0.10 ^a^	3.79 ± 0.32 ^b^	2.39 ± 0.35 ^a^
VOO-2	10.97 ± 0.42	4.72 ± 0.09 ^a^	7.33 ± 0.23 ^a^	3.80 ± 0.10 ^b^	5.60 ± 0.18 ^b^
VOO-3	5.96 ± 0.42	3.41 ± 0.45 ^a^	4.15 ± 0.41 ^a^	2.45 ± 0.18 ^b^	2.99 ± 0.21 ^b^
VOO-9	2.18 ± 0.14	2.38 ± 0.01 ^a^	3.64 ± 0.06 ^a^	2.06 ± 0.02 ^b^	3.27 ± 0.08 ^b^
VOO-10	3.41 ± 0.24	3.28 ± 0.01 ^a^	4.47 ± 0.03 ^a^	3.19 ± 0.03 ^b^	4.34 ± 0.04 ^b^
VOO-11	3.17 ± 0.04	2.34 ± 0.00 ^b^	3.37 ± 0.01 ^b^	2.90 ± 0.01 ^a^	3.91 ± 0.19 ^a^
VOO-12	2.85 ± 0.20	2.69 ± 0.04 ^b^	5.08 ± 0.01 ^b^	3.25 ± 0.19 ^a^	5.80 ± 0.20 ^a^
VOO-13	3.52 ± 0.07	2.65 ± 0.07 ^a^	2.98 ± 0.02 ^a^	2.25 ± 0.06 ^b^	2.84 ± 0.29 ^a^

Values are means ± standard deviation; *n* = 3; Values bearing different lowercase letters as superscripts for each sample and the same analyte determined by the two techniques are statistically different at *p* < 0.05 (paired *t*-test).

**Table 6:** Determination of total Hytr, total Tyr content (mg/20 g oil) of VOO samples analyzed by UHPLC-DAD and HPLC-DAD.

	F-C Assay	UHPLC-DAD		HPLC-DAD	
Samples	Total Phenol Content	Total Htyr Content	Total Tyr Content	Total Htyr Content	Total Tyr Content
	(mg CA/20 g Oil)	(mg/20 g Oil)			
VOO-1	4.74 ± 0.47	4.84 ± 0.22 ^a^	2.78 ± 0.18 ^a^	4.05 ± 0.05 ^b^	2.39 ± 0.04 ^a^
VOO-2	10.97 ± 0.42	4.48 ± 0.16 ^a^	7.73 ± 0.40 ^a^	4.10 ± 0.20 ^b^	5.95 ± 0.15 ^b^
VOO-3	5.96 ± 0.42	3.64 ± 0.02 ^a^	4.72 ± 0.10 ^a^	3.29 ± 0.65 ^a^	3.84 ± 0.30 ^b^
VOO-9	2.18 ± 0.14	4.42 ± 0.01 ^a^	5.73 ± 0.03 ^b^	3.73 ± 0.05 ^b^	5.95 ± 0.14 ^a^
VOO-10	3.41 ± 0.24	3.11 ± 0.01 ^b^	4.43 ± 0.05 ^b^	5.87 ± 0.14 ^a^	7.71 ± 0.18 ^a^
VOO-11	3.17 ± 0.04	2.90 ± 0.01 ^b^	4.34 ± 0.03 ^b^	3.95 ± 0.09 ^a^	4.77 ± 0.23 ^a^
VOO-12	2.85 ± 0.20	3.68 ± 0.03 ^a^	5.79 ± 0.03 ^b^	4.68 ± 0.02 ^b^	7.87 ± 0.04 ^a^
VOO-13	3.52 ± 0.07	3.61 ± 0.02 ^a^	3.95 ± 0.03 ^b^	3.85 ± 0.19 ^a^	4.32 ± 0.18 ^a^

Extraction was carried out according to Mastralexi et al. [19] using as extraction solvent methanol:water, 80:20 *v*/*v*. Values are means ± standard deviation (*n* = 3); Values bearing different lowercase letters as superscripts for each sample and the same analyte determined by the two techniques are statistically different at *p* < 0.05 (paired *t*-test).

## Abbreviations

ANOVA	Analysis of variance
AOAC	Association of Official Agricultural Chemists
CA	caffeic acid
DHPA	3,4-dihydrophenylacetic acid
DAD	diode array detector
EU	European Union
F-C	Folin-Ciocalteu
FLD	fluorescence detector
HPLC	high performance liquid chromatography
HPLC-DAD	high performance liquid chromatography coupled to diode array detector
Htyr	Hydroxytyrosol
ICH	International Conference on Harmonization
IOC	International Olive Council
LOD	limit of detection
LOQ	limit of quantification
MS	mean square
PF	polar fraction
ROO	refined olive oil
RSD	relative standard deviation
SD	standard deviation
MS/MS	tandem mass spectrometry
SS	sum of squares
Tyr	Tyrosol
UHPLC	ultra-high performance liquid chromatography
UHPLC-MS	ultra-high performance liquid chromatography coupled to mass spectrometry
UV	ultraviolet
USDA	United States Department of Agriculture
VOO	virgin olive oil
